# Beclin 1 over- and underexpression in colorectal cancer: distinct patterns relate to prognosis and tumour hypoxia

**DOI:** 10.1038/sj.bjc.6605904

**Published:** 2010-09-14

**Authors:** M I Koukourakis, A Giatromanolaki, E Sivridis, M Pitiakoudis, K C Gatter, A L Harris

**Affiliations:** 1 Department of Radiotherapy/Oncology, Democritus University of Thrace and University General Hospital of Alexandroupolis, Alexandroupolis, 68100, Greece; 2 Department of Pathology, Democritus University of Thrace and University General Hospital of Alexandroupolis, Alexandroupolis, 68100, Greece; 3 Department of Surgery, Democritus University of Thrace and University General Hospital of Alexandroupolis, Alexandroupolis, 68100, Greece; 4 Cancer Research UK, Molecular Oncology Laboratories, Weatherall Institute of Molecular Medicine and Nuffield Department of Clinical Laboratory Science, University of Oxford, John Radcliffe Hospital, Oxford, OX3 7LJ, UK

**Keywords:** Beclin 1, colon cancer, autophagy, HIF1*α*, LDH

## Abstract

**Introduction:**

Autophagy enables cells to recycle long-lived proteins or damaged organelles. Beclin 1, the mammalian orthologue of the yeast *Apg6/Vps30* gene, functions as a scaffold for the formation of autophagosomes.

**Materials and method:**

The immunohistochemical patterns of Beclin 1 expression and their prognostic relevance were studied in formalin-fixed tissues from 155 patients with colorectal adenocarcinoma treated with surgery alone.

**Results:**

Using the weak homogeneous expression of Beclin 1 in normal colonic tissues as a basis for assessing tumours, the following grouping/staining patterns were recognised in colorectal carcinomas: a normal-like pattern in 62 of 155 (40%) cases, an underexpression pattern in 24 of 155 (15.5%) cases, extensive overexpression of Beclin 1 in 33 of 155 (21.3%) tumours and limited overexpression of the protein in 36 of 155 (23.2%) tumours. Extensive overexpression of Beclin 1 was significantly linked with overexpression of HIF1*α* and LDH5, as well as with high histological grade, vascular invasion and nodal involvement. Furthermore, patients with extensive over- or underexpression of Beclin 1 had a significantly poorer overall survival compared with the other two groups (*P*<0.0001). Beclin 1 had an independent prognostic relevance in multivariate analysis.

**Conclusions:**

Beclin 1 has an important role in growth and metastasis of colorectal cancer. Loss of Beclin 1 expression (allelic loss or microRNA regulatory activity, as suggested in the literature) defines poor prognosis presumably by promoting anti-apoptotic pathways, while overexpression of the protein, being linked with tumour hypoxia and acidity, also defines subgroups of tumours with aggressive clinical behaviour.

Autophagy is an important cellular function that enables the recycling of long-lived proteins or damaged organelles (Klionsky and Emr, 2000). Autophagy is characterised by the formation of double-membrane vacuoles containing cytoplasmic constituents, the autophagosomes. These are fused with lysosomes, which subsequently degrade the sequestered material. In this way, the cellular homoeostasis is preserved by preventing the accumulation of waste material, which, at the same time, is degraded and used as metabolic fuel. Various stress factors, including hypoxia, acidity and starvation, accentuate the autophagic machinery. Oncogenes and tumour suppressor genes are also involved in the regulation of autophagy (Maiuri *et al*, 2009).

Cancer cells are, therefore, exposed to a variety of external and internal stimuli that would intensify their autophagic activity. The role of autophagy in the growth and metastasis of human carcinomas is poorly understood. Although autophagy is a major cell survival pathway, excessive activation leads to massive degradation of cellular components, shifting the balance to self-destruction and autophagic cell death (type II programmed cell death). The autophagic cancer cell response to ionising radiation and chemotherapy seems to affect the efficacy of these agents, but such interactions are very complex depending on the agent and the cell type studied (Zois and Koukourakis, 2009).

The core machinery of autophagy is composed of three major functional groups: (i) Atg9 and its cycling system; (ii) a phosphatidylinositol 3-OH kinase (PI3K) complex; and (iii) an ubiquitin-like protein system, which includes two ubiquitin proteins, the Atg12 and Atg8 (mammalian homologue MAP1LC3/LC3) (Mari and Reggiori, 2007). In mammalian cells, the class III PI3K complex is composed of various interacting proteins (UVRAG, Bif-1 and Ambra-1) and the vacuolar protein-sorting proteins Vps34, Vps15 and Beclin 1/Atg6. Beclin 1 is the mammalian orthologue of the yeast *Apg6/Vps30* gene (Cao and Klionsky, 2007). It functions as a scaffold for the formation of the PI3K complex, which is one of the first components recruited by a developing autophagosome, and is essential for autophagy. Beclin 1 is present in intra-cytoplasmic organelles, such as the *trans*-Golgi network, endoplasmic reticulum, mitochondria and the perinuclear membrane (Kihara *et al*, 2001; Cao and Klionsky, 2007).

In this study, we assessed the patterns of Beclin 1 expression in a series of colorectal carcinomas with scope to investigate the clinicopathological role of this protein in the early steps of autophagosome formation.

## Materials and methods

The material comprised formalin-fixed paraffin-embedded tissues from 155 consecutive patients with operable stage II/III colorectal adenocarcinoma treated with surgery alone. This is a retrospective study based on archival material. The cases analysed represent a sequential series of patients according to the archival number given at the Department of Cellular Pathology, Oxford, UK, upon receipt of the surgical specimen. The selection of this specific material was performed to include patients who underwent surgery alone without chemotherapy of radiotherapy at a time when these adjunctive therapies were not the standard of treatment. In this way, the analysis of data in this older series will reflect the actual impact of the tumour biology on the clinical outcome, without introducing into our model unpredictable differences in terms of chemo- or radiosensitivity of individual carcinomas, which would then need randomised comparison to interpret. All patients in the series underwent surgery between 1990 and 1994 having a follow-up of at least 3 years after surgery. Patients with perioperative death were excluded from this study.

According to the TNM AJCC/UICC staging system, 80 cases were stage IIA (T3N0) and 75 were stage III (T3-N1,2). Altogether, 49 of the 155 patients had tumours with rectal involvement. In all, 66 of them were female and 89 were male. The median age was 69 (range 37–87) years. The median follow-up was 24 (range 3–43) months.

### Immunohistochemistry

For the detecton of Beclin 1, the rabbit monoclonal antibody EPR1733Y (ab51031, Abcam, Cambridge, UK) was used at a dilution 1 : 50. This is a synthetic peptide corresponding to residues near the C-terminus of human Beclin 1. The concentration of the aforementioned antibody was determined after performing a pilot study in which the dilutions of the primary antibody varied from 1 : 10 to 1 : 400, and the incubation time was 75 min at room temperature *vs* overnight incubation at 4°C.

Sections were cut at 3 *μ*m and stained as follows: They were dewaxed and rehydrated in graded alcohol solutions. For heat-induced epitope retrieval, the sections were placed in citrate buffer (1 : 10 dilution, pH 7.2) and heated at 120°C for 3 × 5 min. Endogenous peroxidase activity was neutralised using Peroxidase Block for 5 min. The nonspecific binding was blocked by pre-incubation with Protein Block for 5 min at room temperature (Novocastra Laboratories Ltd., Newcastle upon Tyne, UK). Slides were then incubated overnight at 4°C with the primary antibody diluted 1 : 50 (Abgent, San Diego, CA, USA). The slides were washed with PBS (2 × 5 min) and then incubated with Post Primary Block (that enhances penetration of the subsequent polymer reagent) for 30 min at room temperature (Novocastra Laboratories Ltd.), and then washed with PBS for 2 × 5 min and incubated with NovoLink polymer for 30 min at room temperature (Novocastra Laboratories Ltd.). This recognises mouse and rabbit immunoglobulins and detects any tissue-bound primary antibody. After extensive washing with PBS (2 × 5 min), the colour reaction was developed in 3,3′-diaminobenzidine for 5 min. The sections were then counterstained with hematoxylin, dehydrated and mounted.

Normal rabbit immunoglobulin G was substituted for the primary antibody as negative control. Staining with omission of the primary antibody was also performed as negative control.

### Assessment of Beclin 1 expression

Beclin 1 was expressed in both the nuclei and cytoplasm of cancer cells. The percentage of cells with positive nuclei was recorded in all available optical fields at × 200 magnification, and the mean value was used to score each case. The median value of these was used to group cases into two categories of low (<median) and high (⩾median) nuclear reactivity.

**Figure 1:**
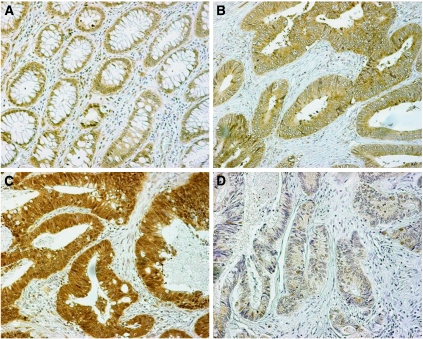
Immunohistochemical patterns of Beclin 1 expression: (**A**) Normal colonic tissue showing weak cytoplasmic expression of Beclin 1 in parallel with sporadic staining of nuclei. (**B**) Normal-like Beclin 1 expression pattern in a colorectal adenocarcinoma. (**C**) Overexpression of Beclin 1 in the cytoplasm and nuclei of a colorectal adenocarcinoma. (**D**) Downregulation of Beclin 1 expression in a colorectal adenocarcinoma with sporadic weak staining of nuclei.

The intensity and extent of cytoplasmic expression was also recorded in all optical fields. The intensity ranged from negative through weak to strong. The whole tissue section, including tumour and adjacent normal colon area, was examined in all × 200 optical fields available. Given that the normal colonic epithelium consistently showed a weak cytoplasmic expression of Beclin 1, this grade of intensity in tumour cells was considered as ‘normal-like’ (Figure 1A). Taking this weak staining as a criterion, there were tumour cells with loss of Beclin 1 expression and those overexpressing the protein. The percentage of cells with normal-like intensity and those with under- or overexpression was recorded in all × 200 optical fields and the mean value was used to score each case. Combining the results of extent and intensity of staining, the tumours were allocated to the following four categories:

1. ‘Normal-like’ pattern of staining: <10% of the neoplastic cells expressed a strong, and >90% expressed a weak cytoplasmic Beclin 1 reactivity (Figure 1B).
2. Overexpression of Beclin 1: when >10% showed a strong cytoplasmic expression; this was considered as being (2A) extensive when >50% of neoplastic cells showed a strong Beclin 1 reactivity, or (2B) limited when 10–50% of tumour cells showed a similar reaction (Figure 1C). In the 2A pattern, the strong cytoplasmic expression was present in the context of a prevalent weak expression in the tissue sample, whereas in the 2B the strong cytoplasmic expression was the prevalent pattern. It was therefore postulated that such a classification would be important to identify divergent biological behaviour.
3. Underexpression of Beclin 1: >50% of tumour cells were negative (Figure 1D).

In all cases (normal colonic tissues, four tumour categories of immunohistochemical expression), sporadic nuclear expression of Beclin 1 is also evident in parallel with the cytoplasmic staining of cells.

### Assessment of hypoxia/acidity

The presence of hypoxia and acidity was assessed immunohistochemically by detecting the expression of HIF1*α* and LDH5. The HIF1*α* is a key transcription factor stabilised under hypoxic conditions because of the the reduction of its degradation rate by the ubiquitin-proteasome pathway (Salceda and Caro, 1997). LDH5, which is composed of five LDH-A subunits, is the LDH isoenzyme with the highest affinity to catalyse the anaerobic transformation of pyruvate to lactate that is extruded outside the cells through the activity of mocoarboxylate transporters, acidifying the extracellular milieu (Holbrook *et al*, 1975).

HIF1*α* was detected after using the ESEE 122 monoclonal antibody (University of Oxford), whereas the detection of LDH5 was achieved by the ab9002 polyclonal antibody (Abcam). The immunohistochemical technique and the scoring system for these proteins has been previously reported (Koukourakis *et al*, 2006). Cases were grouped into categories of low and high expression, using a previously reported grading system (Koukourakis *et al*, 2006).

### Assessment of vascular invasion and necrosis

The presence of cancer cells within vascular channels was detected in hematoxylin–eosin sections. Areas of necrosis covering >10% of the tissue section was scored as ‘extensive’, whereas all other cases were scored as being of ‘limited/no’ necrosis.

### Statistical analysis

Statistical analysis was performed using the GraphPad Prism 5.0 and the Instat 3.1 package (GraphPad Software Inc., San Diego, CA, USA). A Fisher's exact test was used for testing relationships between categorical variables (contingency tables) as appropriate. Linear regression analysis was used to assess correlation with continuous variables. The Kaplan–Meier survival curves were used to assess the impact of various variables in the overall survival (OS) of patients. A Cox proportional hazard model was used to assess the effect of assessed parameters on death events. A *P*-value of <0.05 was used for significance.

## Results

### Expression patterns

Normal colon area showed invariably a weak Beclin 1 staining of epithelial cells (Figure 1A), and this intensity was considered as normal-like for the subsequent scoring of cancer areas.

The typical patterns of Beclin 1expression (negative, weak cytoplasmic, strong cytoplasmic and nuclear) are shown in Figure 1B–D. The median percentage of cells with strong cytoplasmic expression was 10% (range 0–90%), whereas that for nuclear expression was 0% (range 0–80%).

Using the scoring system described in the Material and Methods section, 33 of 155 (21.3%) cases had extensive overexpression of Beclin 1, 36 of 155 (23.2%) showed limited overexpression, 62 of 155 (40%) showed a normal-like pattern and 24 of 155 (15.5%) were underexpressed. Of the 155 cases, 67 (43.2%) showed nuclear expression of Beclin 1 in ⩾10% of cancer cells

Analysis of nuclear expression according to the cytoplasmic patterns showed that Beclin 1 overexpression (limited and extensive) was linked significantly with nuclear expression (Table 1; *P*<0.02).

### Association with histological variables

The association of cytoplasmic Beclin 1 expression patterns with histopathological variables is shown in Table 2. Extensive Beclin 1 overexpression was significantly linked with nodal involvement, high histological grade and vascular invasion.

High nuclear expression was significantly linked with high histological grade (51 of 67 *vs* 54 of 88; *P*=0.05), and there was a similar trend for such an association with necrosis (49 of 67 *vs* 52 of 88; *P*=0.08).

### Association with hypoxia parameters

Table 2 shows the association of Beclin 1 with HIF1*α* and LDH5 expression. Extensive overexpression of Beclin 1 was significantly linked with high LDH5 expression compared with all other subgroups (*P*<0.01). Both limited and extensive overexpression were linked with high HIF1*α* expression.

### Survival analysis

**Figure 2:**
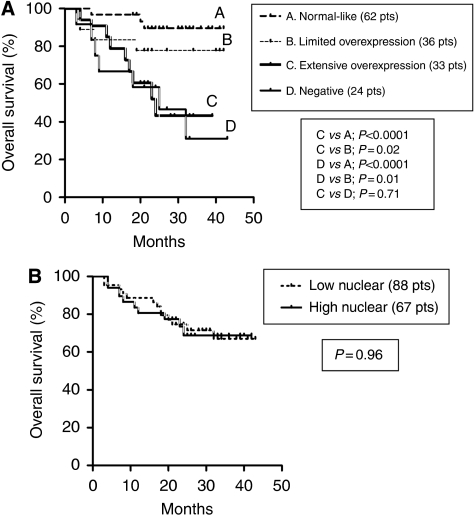
Kaplan–Meier overall survival curves according to the cytoplasmic patterns (**A**) and the nuclear expression (**B**). Pts, patients.

Figure 2A shows Kaplan–Meier disease-specific OS curves, stratified according to the cytoplasmic expression patterns. Patients with extensive overexpression and those with underexpression of Beclin 1 had a significantly poorer OS compared with other two groups (*P*<0.0001). The 3-year OS was 89%, 77.7%, 43.3% and 31% for patients exhibiting normal-like, limited overexpression, extensive overexpression and underexpression patterns, respectively. The nuclear expression of Beclin 1 was not related to prognosis (Figure 2B).

As Beclin 1 showed various associations with histopathological variables known to affect prognosis (stage, differentiation and necrosis), a multivariate analysis was performed to assess the independence of Beclin 1 prognosis. It was shown that the cytoplasmic patterns of Beclin 1, together with those of nodal involvement and vascular invasion, were strong independent predictors of death events (model 1; Table 3). In another model (model 2), which, in addition to the above parameters, the HIF1*α* and LDH5 were included, the LDH5 expression was emerged as an independent prognostic marker.

Despite the low number of cases, double stratification Kaplan–Meier survival analysis, according to Beclin 1 and stage, showed that Beclin 1 patterns of expression maintained their prognostic relevance separately in stage II and in stage III subgroups (data not shown).

## Discussion

The role of the autophagy in the clinical outcome of human malignancies remains obscure. Most of studies focus on Beclin 1, an essential protein for the initiation of autophagosome formation (Cao and Klionsky, 2007). It serves as a scaffold, which, by binding to other proteins, forms pre-autophagosomal structures. Beclin 1 is reported to reside in the *trans*-Golgi network, endoplasmic reticulum and the mitochondria (Kihara *et al*, 2001; Cao and Klionsky, 2007). It is therefore expected that Beclin 1 will show a cytoplasmic patterns of immunohistochemical expression. This was, indeed, confirmed in our study, although an additional nuclear localisation of the protein was also noted in a subset of colorectal tumours. The latter pattern has been also reported by Li *et al* (2009) in a series of colon carcinomas. Beclin 1 contains a leucine-rich nuclear export signal motif, and Liang *et al* (2001) showed that the CRM1 nuclear export pathway is an important regulator of Beclin 1 autophagic and tumour suppression function.

The existing data on the prognostic role of Beclin 1 in human carcinomas are rather scarce and contradictory. Ahn *et al* (2007) reported that Beclin 1 is poorly expressed in normal colon and gastric mucosa, a finding that is in accordance with the current study. Overexpression of Beclin 1 in related carcinomas was a very frequent event and the authors suggested that inactivation or loss of Beclin 1 expression may not occur in gastrointestinal carcinomas (Ahn *et al*, 2007). Li *et al* found that higher levels of Beclin 1 expression are linked with better prognosis in patients with stage III colorectal cancer (Liang *et al*, 2001). A study in hepatocellular carcinomas, on the other hand, suggested that the loss of Beclin 1 in tumour cells was the most prominent expression pattern, which was also related to recurrent disease (Ding *et al*, 2008). Liang *et al* (1999) found a relative underexpression of Beclin 1 in breast carcinomas compared with normal breast tissues, suggesting that decreased expression of autophagic proteins may contribute to the development or progression of breast cancer. Beclin 1 levels are decreased in high-grade brain tumours and, high Beclin 1 levels linked with better prognosis (Miracco *et al*, 2007; Pirtoli *et al*, 2009). Indeed, allelic deletions of Beclin 1 are common in breast cancer cell lines and promote tumourigenesis in experimental models (Qu *et al*, 2003). Moreover, microRNAs, such as miT-30a, seem also to be involved in the negative regulation of Beclin 1 (Zhu *et al*, 2009).

Therefore, it appears that both under- and overexpression of Beclin 1 may exist in human carcinomas. Indeed, in our study, by taking the intensity of normal colonic staining as a marker for tumour reactivity, we managed to identify four distinct patterns of Beclin 1 expression: underexpression of Beclin 1, which was evident in 15.5% of tumours, the normal-like pattern, which was present in 40.6%, and overexpression of Beclin 1, which was detected in the remaining 43.9% of cases. This latter category, however, was further split into two equal-numbered subgroups of limited and extensive overexpression. Extensive overexpression of Beclin 1 was significantly linked with histological markers of tumour aggressiveness, such as nodal involvement, high histological grade and vascular invasion. Survival analysis confirmed the ominous prognostic role of extensive Beclin 1 overexpression, but a similarly poor survival was also showed in tumours with loss of Beclin 1 expression.

The above analysis suggests the existence of two distinct biological pathways of Beclin 1 activity, both linked with tumour aggressiveness. Loss of Beclin 1 expression may reflect allelic gene deletions (Aita *et al*, 1999). As Beclin 1 interacts with members of the bcl-2 protein acting as a tumour suppressor (Cao and Klionsky, 2007), potentiation of the anti-apoptotic machinery may account, at least in part, for the poor outcome of patients having Beclin1-negative colonic tumours and would not necessarily be related to severe environmental stress.

Maintenance of Beclin1 expression, however, may not necessarily be associated with good prognosis. Overexpression of the protein, often linked with its nuclear presence, especially when extensively present in tumours, also defined poor prognosis. However, nuclear expression *per se* was not linked with prognosis, suggesting that the cytoplasmic presence of Beclin 1 is more potent marker of aggressiveness. If the nuclear presence also has a biological relevance, this was masked by the prominent prognostic impact of the cytoplasmic overexpression. Although a direct involvement of an activated autophagic machinery in cancer cell survival and growth cannot be excluded, Beclin 1 overexpression may be an indirect marker of tumour aggressiveness. Extensive overexpression was linked with HIF1*α* and LDH5 upregulation, indicating hypoxic tumours and an acidic intra-tumoral environment. Chen *et al* (2009) also found a direct association between Beclin 1 and HIF1*α* expression in oesophageal carcinoma. Upregulation of Beclin 1 may be a response to adverse intra-tumoral conditions demanding intensified autophagic activity for cancer cells to recycle proteins and damaged organelles in order to survive. Samokhvalov *et al* (2008) showed that RNAi knockdown of Beclin 1 function decreases survival under hypoxic stress. Overexpression of Beclin 1 may therefore be a survival response to adverse intra-tumoral conditions and could indicate tumour aggressiveness linked with hypoxia and acidity. In the current study, multivariate analysis of parameters, including HIF1*α* and LDH5, showed that Beclin 1 had an independent prognostic relevance so that a direct effect of Beclin 1 in defining tumour aggressiveness is implied, aside to its association with hypoxia and acidity.

The current series of patients, being treated with surgery alone, has been intentionally chosen to study the biological role of Beclin 1 in the clinical course of the disease, avoiding bias from interactions of chemotherapy or radiotherapy with the autophagic machinery. Whether the specific Beclin 1 expression patterns further define responsiveness to such therapies remains an important question to answer in subsequent studies in randomised series of patients receiving postoperative chemo- or radiotherapy. Nevertheless, from a therapeutic point of view, Beclin 1 emerges as a target for development of molecular therapies. Silencing the Beclin 1 protein may prove lethal for tumours whose survival relies on intensified autophagic cell response, such as tumours with high hypoxic cell content. Song *et al* (2009) found that treatment of hepatoma cells with Beclin 1 siRNA resulted in chemosensitisation under hypoxic conditions. Blocked autophagy also sensitises cancer cells to radiation (Apel *et al*, 2008). In a recent study, Kim *et al* (2008) showed that Beclin 1 is linked with HIF1*α* expression and poor survival of nasopharyngeal cancer patients undergoing radiotherapy and chemotherapy. In a contrary study, tumours with repressed Beclin 1 expression seem to bear increased inherent radioresistance because of increased anti-apoptotic potential, so that targeting the bcl-2 or p53 pathway may be a suitable means for radiosensitisation (Anai *et al*, 2007).

It is concluded that Beclin 1 has an important role in growth and metastasis of human colorectal cancer. Loss of Beclin 1 expression, presumably related to allelic loss of the gene or microRNA regulatory activity as suggested in the literature, defines poor prognosis by promoting anti-apoptotic pathways, whereas overexpression of the protein, being linked with tumour hypoxia and acidity, also defines subgroups of tumours with an aggressive clinical behaviour. Elucidating the autophagy mechanisms in cancer cells and careful classification of patients may prove of importance in the development of such targeted therapeutic strategies.

## Figures and Tables

**Table 1:** Relation of cytoplasmic patterns of Beclin 1 to nuclear expression

	**Nuclear expression**		
**Cytoplasmic patterns**	**0–9%**	**⩾10%**	***P*-value**
1 – Normal-like	41	22	2 *vs* 1; 0.02
2A – Limited overexpression	14	22	2 *vs* 3; 0.01
2B – Extensive overexpression	16	16	—
3 – Underexpression	18	6	—

**Table 2:** Relation of cytoplasmic Beclin 1 expression patterns to histopathological and hypoxia parameters

	**Cytoplasmic patterns**				
**Parameter**	**Normal- like**	**Limited overexpression**	**Extensive overexpression**	**Underexpression**	***P*-value**
*Stage*					
T3-N0	34	20	12	14	0.04^a^
T3-N1,2	28	16	21	10	—
					
*Grade*					
1	26	12	4	8	<0.05^b^
2/3	36	24	29	16	—
					
*Necrosis*					
No	22	6	14	12	<0.05^c^
Yes	40	30	19	12	—
					
*Vascular invasion*					
No	50	26	20	16	0.03^d^
Yes	12	10	13	8	—
					
*HIF1α*					
Low	26	6	7	12	<0.05^e^
High	36	30	26	12	—
					
*LDH5*					
Low	20	12	2	8	<0.01^f^
High	42	24	31	16	—

a Extensive *vs* all other.

b Extensive *vs* each one of the other groups.

c Limited *vs* each one of the other groups.

d Extensive *vs* normal.

e Extensive and limited overexpression *vs* each one of the rest.

f Extensive overexpression *vs* each one of the rest.

**Table 3:** Multivariate analysis of death events (model 1: Beclin 1 and histopathological variables; model 2: significant variables of model 1 including hypoxia variables)

	**Model 1**		**Model 2**	
**Variable**	***t* ratio**	***P*-value**	***t* ratio**	***P*-value**
Nodal involvement	4.04	<0.0001	4.89	<0.0001
Grade (2/3 *vs* 1)	0.09	0.92	—	—
Vascular (yes *vs* no)	3.13	0.002	2.71	0.007
Necrosis (yeas *vs* no)	0.03	0.30	—	—
Beclin 1cytoplasmic	5.05	<0.0001	4.97	<0.0001
Beclin 1 nuclear	0.08	0.93	—	—
LDH5	—	—	4.18	<0.0001
HIF1*α*	—	—	0.69	0.48
